# Cardiovascular and Metabolic Responses to the Ingestion of Caffeinated Herbal Tea: Drink It Hot or Cold?

**DOI:** 10.3389/fphys.2018.00315

**Published:** 2018-04-06

**Authors:** Claire Maufrais, Delphine Sarafian, Abdul Dulloo, Jean-Pierre Montani

**Affiliations:** Laboratory of Integrative Cardiovascular and Metabolic Physiology, Division of Physiology, Department of Medicine, University of Fribourg, Fribourg, Switzerland

**Keywords:** caffeinated herbal tea, drink temperature, energy expenditure, hemodynamics, cutaneous blood flow

## Abstract

**Aim:** Tea is usually consumed at two temperatures (as hot tea or as iced tea). However, the importance of drink temperature on the cardiovascular system and on metabolism has not been thoroughly investigated. The purpose of this study was to compare the cardiovascular, metabolic and cutaneous responses to the ingestion of caffeinated herbal tea (Yerba Mate) at cold or hot temperature in healthy young subjects. We hypothesized that ingestion of cold tea induces a higher increase in energy expenditure than hot tea without eliciting any negative effects on the cardiovascular system.

**Methods:** Cardiovascular, metabolic and cutaneous responses were analyzed in 23 healthy subjects (12 men and 11 women) sitting comfortably during a 30-min baseline and 90 min following the ingestion of 500 mL of an unsweetened Yerba Mate tea ingested over 5 min either at cold (~3°C) or hot (~55°C) temperature, according to a randomized cross-over design.

**Results:** Averaged over the 90 min post-drink ingestion and compared to hot tea, cold tea induced (1) a decrease in heart rate (cold tea: −5 ± 1 beats.min^−1^; hot tea: −1 ± 1 beats.min^−1^, *p* < 0.05), double product, skin blood flow and hand temperature and (2) an increase in baroreflex sensitivity, fat oxidation and energy expenditure (cold tea: +8.3%; hot tea: +3.7%, *p* < 0.05). Averaged over the 90 min post-drink ingestion, we observed no differences of tea temperature on cardiac output work and mean blood pressure responses.

**Conclusion:** Ingestion of an unsweetened caffeinated herbal tea at cold temperature induced a greater stimulation of thermogenesis and fat oxidation than hot tea while decreasing cardiac load as suggested by the decrease in the double product. Further experiments are needed to evaluate the clinical impact of unsweetened caffeinated herbal tea at a cold temperature for weight control.

## Introduction

Tea is the second most commonly consumed beverage in the world after water (Macfarlane and Macfarlane, 2004). Tea is usually consumed at two temperatures (as hot tea or as iced tea). However, the importance of drink temperature on the cardiovascular system and on metabolism has not been thoroughly investigated. An epidemiological study (Vernarelli and Lambert, 2013) has compared the metabolic health outcomes of tea consumption in a representative sample of 5,948 adults (54% female) from the 2003 to 2006 National Health and Nutrition Examination Survey (NHANES) data, and concluded that hot tea consumption was negatively correlated with markers of metabolic syndrome, a beneficial effect which was not found in iced tea consumers. However, this comparison between hot and iced tea is somewhat hampered by the fact that this is an epidemiological study in various groups of subjects. Moreover, iced tea is usually consumed in greater amounts and with higher sugar content. A careful study comparing in the same individual hot vs. iced tea, with the same ingredient composition and without the confounding problem of sugar, is thus lacking.

Numerous forms of teas are consumed around the world. Classical tea, made from an infusion of the dried leaves of the tree *Camellia sinensis* and consumed commonly as green, oolong or black tea, is one of the most popular drinks in many populations and contains various concentrations of caffeine (Hicks et al., 1996). In contrast, herbal teas, made out of the infusion or decoction of herbs, spices, fruits and other plants usually do not contain caffeine. However, a common herbal tea, also containing caffeine, is Yerba Mate tea, made from an infusion of the dried leaves of the tree *Ilex paraguariensis*. It is widely consumed in Southern Latin American countries and is gaining rapid penetration into world markets (Heck and De Mejia, 2007). It is not only the outward properties of Yerba Mate tea that distinguish it from teas (i.e., flavor, aroma) but also its diverse concentration of biological compounds that are readily found in teas (Heck and De Mejia, 2007). Most notably, of these compounds are the xanthines (e.g., caffeine, theophylline, and theobromine), which can increase energy expenditure (EE) (Dulloo, 2011), and polyphenols with high antioxidant capacity (Bracesco et al., 2003). Thus, as reported in the review of Heck and De Mejia (2007), Yerba Mate tea has higher antioxidant capacity than both green and black teas, even if green tea is touted as having a very high antioxidant capacity. Many reports using animal models also highlighted vasodilatory, lipid-lowering properties, anti-mutagenic and anti-glycation effects of Yerba Mate ingestion (Bracesco et al., 2003). These properties have often accompanied weight and fat loss and increased energy metabolism as recently demonstrated in rodent studies (Borges et al., 2013). We chose Yerba Mate tea because compared to other stimulant beverages, such as black tea, green tea and coffee, scientific reports relating to its ingestion in humans are scarce. A past paper described that Yerba Mate ingestion induced no changes in heart rate (HR), blood pressure (BP), EE but a reduced respiratory quotient (RQ, i.e., a rise in the proportion of fat oxidized) (Martinet et al., 1999). In contrast, many studies analyzed the effects of green and black tea ingestion on the cardiovascular and metabolic system. Dulloo et al. (1999) found a significant increase in 24-h EE and a reduced RQ after green tea ingestion but no effect on HR. Because Yerba Mate is consumed worldwide, it would be of major interest to provide a comprehensive cardiovascular and metabolic analysis of its ingestion.

Yerba Mate tea is a beverage that can be prepared using not only hot but also with cold water (Bracesco et al., 2011). We are not aware of any study that has evaluated the impact of drink temperature on the Yerba Mate tea ingestion. However, we already demonstrated differential cardiovascular and metabolic responses to the temperature of drinking water [i.e., cold (~3°C) vs. body-tempered (~37°C) water] in young and healthy adults (Girona et al., 2014). For instance, we found an immediate and substantial depression of HR and skin blood flow (SkBf) after ingestion of cold- but not body-tempered water. Moreover, we found an increase in EE after ingestion of cold- but not body-tempered water. The drink temperature effects on EE may be furthered augmented with the introduction of Yerba Mate in the beverage, considering that metabolism is already stimulated with this plant. The increase in EE and substrate oxidation may be of major interest for weight control programs. In this context, the purpose of this study was to evaluate the cardiovascular, metabolic and cutaneous responses to an unsweetened caffeinated herbal tea ingestion at different temperatures in healthy young subjects. We hypothesized that ingestion of cold Yerba Mate tea induces a higher increase in EE than hot tea without any negative effects on the cardiovascular system.

## Materials and methods

### Subjects

Twenty-three healthy subjects (twelve men and eleven women) were recruited from local University students and their friends. The mean age of the participants was 24 ± 1 years and body mass index (BMI) 22.5 ± 0.5 kg.m^−2^. Exclusion criteria included those with a BMI greater than 30 kg.m^−2^ and individuals with a daily exercise workload exceeding 60 min per day. None of the subjects had any diseases or were taking any medication affecting cardiovascular or autonomic regulation. Between 2 and 5 days before first test day, the participants visited the laboratory to complete a questionnaire regarding their lifestyle and medical history, and to familiarize themselves with the experimental procedures and equipment. The study protocol complied with the Declaration of Helsinki and received local ethics committee approval (Commission cantonale d'éthique de la recherche sur l'être humain – CER-VD, protocol 2016-01916). Written informed consent was obtained from each subject.

### Study design

All studies started between 08.00 and 09.00 a.m. in an air-conditioned (20–22°C) laboratory dedicated for human physiological measurements. After voiding the bladder, body weight and height were measured using a mechanical column scale with an integrated stadiometer (Seca model 709, Hamburg, Germany). Body composition was measured using a multi-frequency bioelectrical impedance analysis (Inbody 720, Biospace Co., Ltd, Seoul, Korea). All participants were studied in the morning after an overnight (12-h) fast and they were requested to avoid alcohol or caffeine for at least 24 h prior to the test. Furthermore, to minimize the effect of physical activity on the morning of each test day, participants were requested to use motorized transport instead of walking or cycling to reach the laboratory. The subjects were instructed to take a normal meal on the evening before the experiment. Every subject attended two separate experimental sessions (each session separated at least by 2 days) according to a randomized crossover study. Randomization was performed using a random sequence generator (http://www.random.org/sequences/) where the session order was determined for 23 subjects before the study started. Women were only tested during the follicular phase of their menstrual cycle. The subjects were not allowed to know the order of their sessions in advance and were only aware of the full sequence after the first drink. On arrival at the laboratory, subjects were asked to empty their bladders if necessary and to sit in a comfortable armchair. The cardiovascular and metabolic monitoring equipment was then connected. Throughout the procedures, subjects were permitted to watch neutral documentaries on a flat TV screen set at eye level. Following a period of reaching cardiovascular stability (usually ~10–15 min), a stable baseline recording was made for 30 min. Then, the subjects ingested over 5 min, either 500 mL of cold tea (~3°C) or hot tea (~55°C) with a dose of 3.4 g of instant unsweetened Yerba Mate (Wisdom Natural Brands®, 1203 West San Pedro Street Gilbert, Arizona 85233) according to recommendations, containing 99 mg of caffeine (2,900 mg/100 g). The content of caffeine was measured by high-pressure liquid chromatography with UV-detector by an independent laboratory, the Swiss Qualities Testing Services (SQTS, Courtepin, Switzerland; Report 2017L44001/1). Monitoring continued for another 90 min post-drink ingestion. An extra blanket for thermal comfort was provided on demand if subjects felt a little bit cold after the cold drink. We found in preliminary tests that the ingestion of 500 mL hot tea at a temperature of 55°C is a comfortable temperature when the drink is ingested over a few minutes. This was consistent with the study of (Quinlan et al., 1997), in which subjects ingested 400 mL of tea at 56.4°C within 3 min. The dose of Yerba Mate was chosen to be in line with the suggested serving size. We chose an instant tea but not a tea bag to have the same dose of tea dissolved in water. The Mate tea is also unsweetened to avoid the effects of sugar usually described in the literature (Grasser et al., 2014).

### Cardiovascular recordings

Cardiovascular recordings were performed using a Task Force Monitor (CNSystems, Medizintechnik, Graz, Austria) with data sampled at a rate of 1,000 Hz. Cardiac intervals (and their reciprocal, HR) were recorded by electrocardiography. BP was recorded using oscillometric brachial BP measurements on the left arm. Thoracic impedance was recorded using band electrodes, one placed on the back of the neck and two parallel electrodes placed on the neck and thorax. Cardiac stroke volume (SV) was derived on a beat-to-beat basis from the impedance cardiogram. This method demonstrated good correlations with standard measures of cardiac output (CO) (Wang et al., 2013). High frequency (HF: 0.17–0.40 Hz) power components of RR intervals (HF_RRI) were evaluated and given in absolute values (ms^2^). Keeping in mind the limitations (Parati et al., 2006), we used changes in the HF range of HR variability to assess parasympathetic activity because HF_RRI is primarily mediated by parasympathetic nerve modulation (Pagani et al., 1997; Stauss, 2003). Baroreflex sensitivity (BRS) was determined from spontaneous fluctuations in BP and cardiac interval using the sequence technique (Bertinieri et al., 1985).

### Metabolic parameters

EE and the RQ (both from oxygen consumption and carbon dioxide production) were assessed non-invasively and continuously using a ventilated hood with the Cosmed system (Quark RMR, Cosmed, Rome, Italy) as described previously (Miles-Chan et al., 2015). The hood was temporarily removed during tea ingestion.

### Cutaneous blood flow and skin temperature

SkBf was recorded non-invasively throughout the whole experiment by laser Doppler flowmetry (LDF) (Perimed, Periflux System PF5001, Järfälla, Sweden). The probe of the LDF was set on the dorsum of the left hand between the thumb and the index finger as described previously (Girona et al., 2014).

We made 3 thermographic pictures with FLIR ex (FLIR Systems) of the left hand every 5 min during baseline and the first 10 min post-drink and then every 10 min until the end of the experiment. Skin temperature on the back of the hand was assessed as described previously (Maufrais et al., 2017).

### Data analysis

Values of cardiac interval, systolic BP (SBP), diastolic BP (DBP), SV, SkBf and skin temperatures were averaged every 15 min during the baseline period. Then, these data were averaged from 0 to 10, 10 to 20, 20 to 30, 30 to 45, 45 to 60, 60 to 75, and 75 to 90 min post-drink period. CO was computed as the product of SV and HR, where HR was calculated from the appropriate cardiac interval. Total peripheral resistance (TPR) was calculated as mean BP (MBP) divided by CO, where MBP was calculated as the result of DBP + 1/3 (SBP-DBP). Double (rate pressure) product (DP) was calculated as HR x SBP and provides valuable information for the oxygen consumption of the myocardium (van Vliet and Montani, 1999). Cardiac output work (CW) was calculated as MBP × CO /451 (Fincke et al., 2004). By coupling both pressure (i.e., MBP) and flow (i.e., CO) domains of the cardiovascular system, it is an integrative measure of cardiac pumping power. Cutaneous vascular resistance was calculated as MBP divided by SkBf.

### Statistical analysis

The number of required subjects was determined by power analysis using the Web software (http://www.statisticalsolutions.net/pssZtest_calc.php), based on a physiologically relevant 0.2 kJ.min^−1^ (5%) change in EE and a conservative standard deviation of 0.75 kJ.min^−1^ based on our previous studies (Girona et al., 2014) (type-I error: 0.05; desired power: 0.80). All values in the text, tables and in figures are expressed as mean ± SEM. Statistical analysis was performed using statistical software (Statview version 5.0, SAS Institute Inc, Cary, NC). To test for changes over time from baseline level and to compare mean changes between the drink types, two-way ANOVA for repeated measures with time and treatment (tea temperature) as within-subject factors with post hoc PLSD of Fisher when appropriate. Linear regression analyses were performed to determine the relationships between body weight and MBP, HR, and EE. Statistical significance for all analyses was considered at *p* < 0.05.

## Results

### Cardiovascular responses

**Figure 1:**
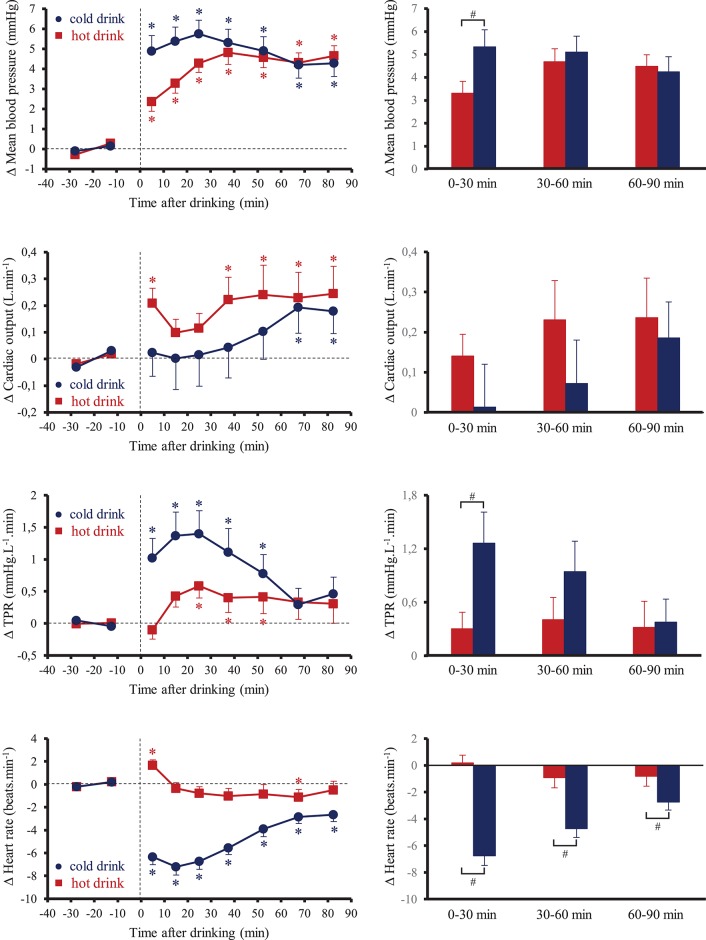
**(Left)** Time course of the changes in mean blood pressure, cardiac output, total peripheral resistance (TPR) and heart rate. **(Right)** Mean responses averaged over 0–30, 30–60, and 60–90 min relative to baseline values and presented as a delta (i.e., average over 0–30, 30–60, and 60–90 min post-drink period, respectively, minus the average over the 30-min baseline period). Drinks: hot tea (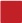 and 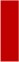); cold tea (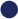 and 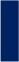). ^*^p < 0.05 significant differences over time from baseline values; ^#^*p* < 0.05 significant differences between responses to the drinks.

Resting baseline values of cardiovascular parameters were not significantly different between the two tests (Table 1). None of the subjects reported any discomfort after the drink was ingested. Figure 1 shows the changes over time for MBP, CO, TPR, and HR. Ingestion of the tea at different temperatures resulted in significant interaction effects (time x tea temperature) for these parameters (*p* < 0.05). Averaged over the first 30 min post-drink ingestion, SBP, MBP, and DBP were higher after cold tea (SBP: +5.2 ± 0.8 mmHg; MBP: +5.4 ± 0.7 mmHg; DBP: +5.4 ± 0.7 mmHg) compared to hot tea (SBP: +3.7 ± 0.7 mmHg; MBP: +3.1 ± 0.4 mmHg; DBP: +2.8 ± 0.5 mmHg; *p* < 0.05). From 30 to 90 min post drink ingestion, we observed similar changes in SBP, MBP, and DBP with both drinks (SBP: cold tea: +4.9 ± 0.7 mmHg; hot tea: +5.2 ± 0.6 mmHg; MBP: cold tea: +4.6 ± 0.5 mmHg; hot tea: +4.6 ± 0.6 mmHg; DBP: cold tea: +4.1 ± 0.6; hot tea: +4.1 ± 0.4 mmHg). When averaged over 90 min post-drink ingestion, SBP, MBP, and DBP were comparable between the two drinks (SBP: cold tea: 5.0 ± 0.7 mmHg; hot tea: 4.7 ± 0.5 mmHg; MBP: cold tea: +4.9 ± 0.6 mmHg; hot tea: +4.1 ± 0.4 mmHg; DBP: cold tea: +4.5 ± 0.6 mmHg; hot tea: +3.7 ± 0.4 mmHg). We observed no statistical differences of tea temperature on CO response averaged over 0–30, 30–60, and 60–90 min post-drink ingestion. Averaged over the first 30 min post-drink ingestion, TPR was higher after cold tea than hot tea (cold tea: +1.2 ± 0.4 mmHg.L^−1^.min; hot tea: +0.3 ± 0.2 mmHg.L^−1^.min, *p* < 0.05) corresponding to an increase of 8 and 1%, respectively. There were no longer significant differences in TPR between cold and hot tea over the following 60 min. Cold tea drinking resulted in an immediate drop in HR, with the largest dip occurring at around 20–30 min (−7 ± 1 beats.min^−1^) and with a gradual attenuation of the bradycardia until the end of the test. Averaged over the 90 min post-drink ingestion, the decrease in HR with cold tea was −5 ± 1 beats.min^−1^. We observed little effects of hot tea drinking on HR relative to baseline.

**Table 1:** Baseline hemodynamic and metabolic data recorded prior to drink ingestion.

	**Hot tea**	**Cold tea**	***p*-value**
Systolic blood pressure (mmHg)	105 ± 2	107 ± 9	NS
Mean blood pressure (mmHg)	80 ± 2	80 ± 1	NS
Diastolic blood pressure (mmHg)	67 ± 2	67 ± 1	NS
Heart rate (beats.min^−1^)	60 ± 2	61 ± 2	NS
Stroke volume (mL)	83 ± 3	83 ± 3	NS
Cardiac output (L.min^−1^)	5.0 ± 0.2	5.0 ± 0.2	NS
Total peripheral resistance (mmHg.L^−1^.min)	16.5 ± 0.6	17.0 ± 1.0	NS
Double product (mmHg.beats.min^−1^)	6, 336±223	6, 512±241	NS
Cardiac output work (watts)	0.88 ± 0.03	0.89 ± 0.03	NS
Baroreflex sensitivity (ms.mmHg^−1^)	27.6 ± 2.5	26.9 ± 2.4	NS
HF_RRI (ln.ms^2^)	2.9 ± 0.1	2.9 ± 0.1	NS
Energy expenditure (KJ.min^−1^)	4.5 ± 0.2	4.5 ± 0.1	NS
Respiratory quotient	0.83 ± 0.01	0.84 ± 0.01	NS
Skin blood flow (AU)	33.8 ± 2.7	33.5 ± 3.2	NS
Cutaneous vascular “resistance” (mmHg/AU)	2.69 ± 0.21	2.83 ± 0.24	NS
Hand temperature (°C)	34.5 ± 0.5	35.0 ± 0.5	NS

*HF_RRI, High frequency power components of RR intervals*.

**Figure 2:**
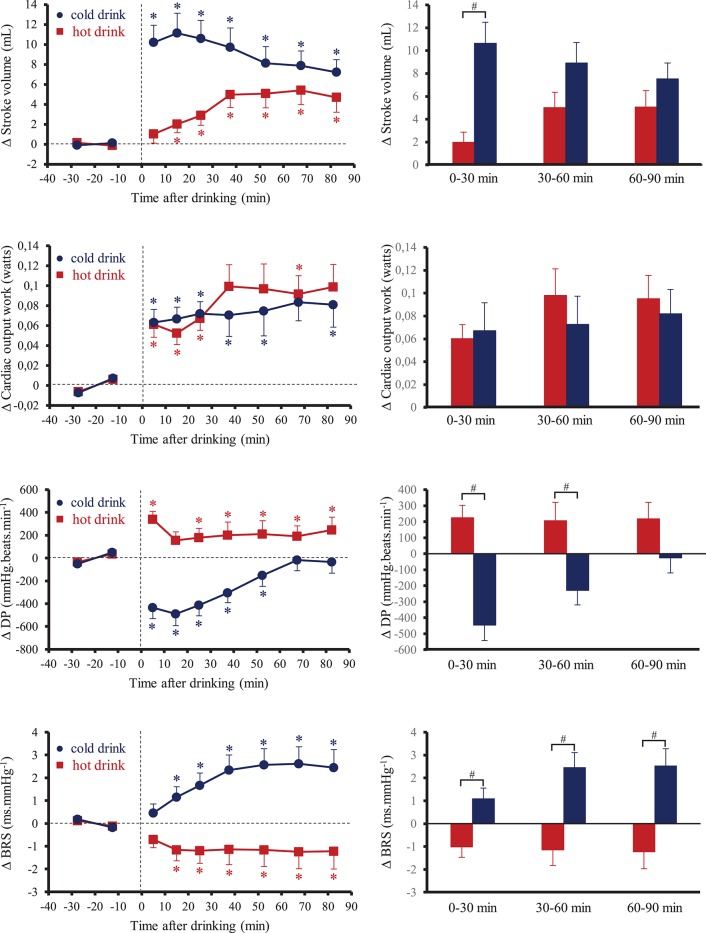
**(Left)** Time course of the changes in stroke volume, cardiac output work, double product (DP) and baroreflex sensitivity (BRS). **(Right)** Mean responses averaged over 0–30, 30–60, and 60–90 min relative to baseline values and presented as a delta (i.e., average over 0–30, 30–60, and 60–90 min post-drink period, respectively, minus the average over the 30-min baseline period). Drinks: hot tea (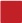 and 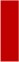); cold tea (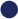 and 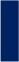). ^*^p < 0.05 significant differences over time from baseline values; ^#^*p* < 0.05 significant differences between responses to the drinks.

Figure 2 shows the changes over time for SV, CW, DP, and BRS. Ingestion of the tea at different temperatures resulted in significant interaction effects (time × tea temperature) for these parameters (*p* < 0.05). Averaging over 60 and 90 min post-drinks periods, the changes in SV were higher after drinking cold tea than hot tea (0–60 min: +10 ± 2 mL vs. +4 ± 1 mL, *p* < 0.05; 0–90 min: +9 ± 1 mL vs. +4 ± 1 mL, *p* < 0.05; cold tea vs. hot tea, respectively). We observed no statistical differences of tea temperature on CW response averaged over 0–30, 30–60, and 60–90 min post-drink ingestion. After drinking hot tea, DP increased and was stable during the 90 min post-drink ingestion. In contrast, cold tea drinking resulted in an immediate drop in DP during the first 20 min post-drink ingestion and gradually returned to baseline values until the end of the test. BRS was higher after drinking cold tea whereas it was lower after drinking hot tea. HF_RRI immediately increased after drinking cold tea at 0–10 min (+7.5 ± 1.6 ln.ms^2^) and then remained relatively elevated until the end of the test (75–90 min: +5.4 ± 1.4 ln.ms^2^). HF_RRI tended to decrease immediately after drinking hot tea (−4.2 ± 1.3 ln.ms^2^), then increased not significantly over baseline levels at 10–20 min (+3.3 ± 1.3 ln.ms^2^) and plateaued until the end of the test.

### Cutaneous and metabolic responses

**Figure 3:**
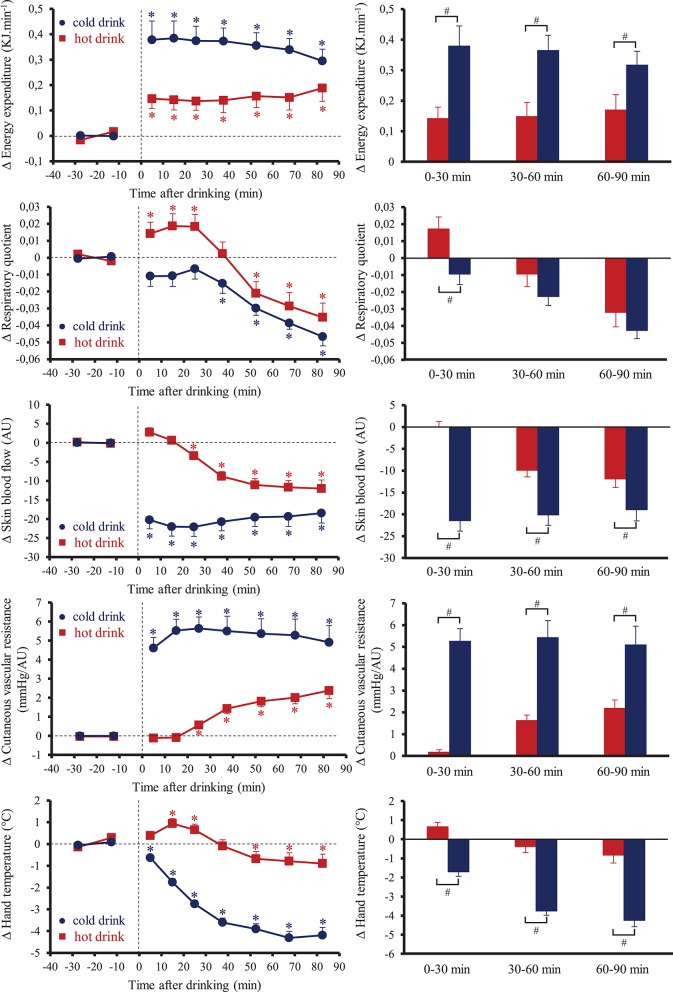
**(Left)** Time course of the changes in energy expenditure (EE), respiratory quotient, skin blood flow, cutaneous vascular resistance, and hand temperature. **(Right)** Mean responses averaged over 0–30, 30–60, and 60–90 min relative to baseline values and presented as a delta (i.e., average over 0–30, 30–60, and 60–90 min post-drink period, respectively, minus the average over the 30-min baseline period). Drinks: hot tea (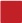 and 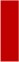); cold tea (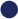 and 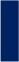). ^*^p < 0.05 significant differences over time from baseline values; ^#^*p* < 0.05 significant differences between responses to the drinks.

Resting baseline values of metabolic parameters were not significantly different between the two tests (Table 1). Figure 3 shows the changes over time for EE, RQ, SkBf, cutaneous vascular resistance and hand temperature. Average over the 90 min post-drink ingestion, EE was higher after cold tea than hot tea (cold tea +0.35 ± 0.05 kJ.min^−1^, +8.3%; hot tea: +0.15 ± 0.04 kJ.min^−1^, +3.7%, *p* < 0.05). During the first 30 min post-drink ingestion, RQ increased with hot tea and tended to decrease with cold tea. Then, RQ decreased with both drinks until the end of the test. Averaged over the 90 min post-drink ingestion, the changes in RQ were lower after drinking cold tea (−0.025 ± 0.004, *p* < 0.05) but not hot tea (−0.008 ± 0.006). SkBf was lower with cold tea than hot tea during the 90 min post-drink ingestion. SkBf decreased immediately after drinking cold tea and plateaued until the end of the test. After drinking hot tea, SkBf progressively decreased from 20 to 30 min until the end of the test but always stayed higher than cold data. Computing cutaneous vascular “resistance,” we observed a greater increase after cold tea than hot tea averaged over the 90 min post-drink ingestion (+5.0 ± 0.7 mmHg/AU vs. 1.0 ± 0.2 mmHg/AU, respectively, *p* < 0.05). Hand temperature progressively decreased after drinking cold tea from 0 to 10 min until the end of the test. We observed small variations of hand temperature after drinking hot tea.

### Gender differences

**Figure 4:**
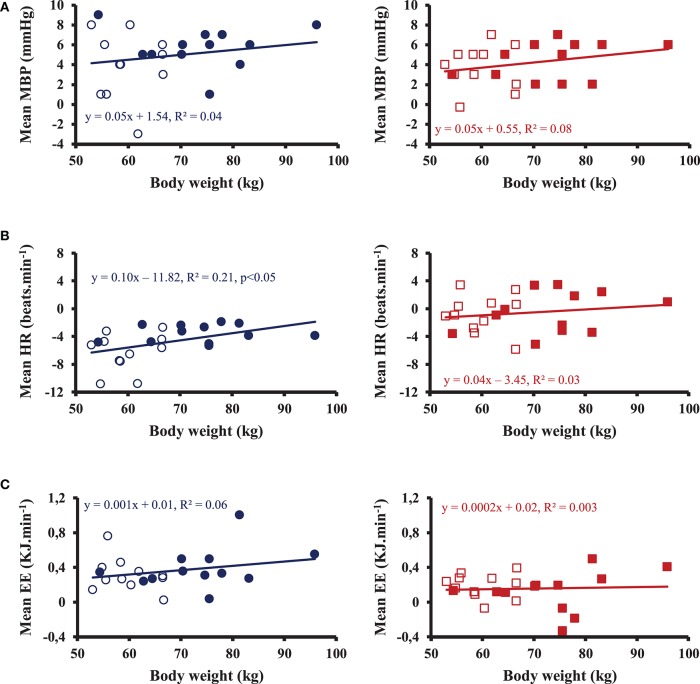
Correlations between mean body weight for 12 men (•, ■) and 11 women (◦, □) and mean blood pressure (MBP) **(A)**, heart rate (HR) **(B)** and energy expenditure (EE) **(C)** averaged over 90 min post-drink ingestion after drinking cold (Left) and hot (Right) Yerba Mate tea.

Averaged over 90 min post-drink ingestion, we observed no gender differences in MBP, CO, TPR, and metabolic responses except a slightly higher BRS, HF_RRI and a tendency for a decrease in HR (*p* = 0.06) in women compared to men with both drinks with no clear interaction with temperature effects. In order to test the impact of body weight on the cardiovascular and metabolic responses with both drinks, we analyzed the correlation between body weight and MBP, HR, and EE, as shown in Figure 4. After hot tea, we did not find correlation between body weight and MBP, HR, and EE. After cold tea, body weight was correlated with mean changes in HR averaged over the 90 min post-drink ingestion, but neither with changes in MBP nor EE.

## Discussion

The aim of this study was to assess the cardiovascular, metabolic and cutaneous responses to an unsweetened caffeinated herbal tea (Yerba Mate) ingestion at different temperatures in healthy young subjects. Our major findings revealed that compared to hot tea, cold tea ingestion induced a slowing down of HR and a decrease in DP associated with a greater EE and fat oxidation.

### Hemodynamic changes with yerba mate tea ingestion

We found an immediate and prolonged depression of HR after ingestion of cold but not hot tea ingestion. This unchanged HR after hot tea was also observed by Martinet et al. (1999). This drop in HR after cold tea ingestion can be explained by alterations in autonomic function. Indeed, we found an increase in BRS and HF_RRI after drinking cold but not hot tea indicating an enhanced cardiac vagal tone drink temperature-related. We already reported that ingestion of 500 mL of cold water leads to an activation of vagal tone (Girona et al., 2014). Ingestion of water below a certain intra-abdominal temperature level raises the cardiac vagal tone which results in a declining HR. It is possible that our observed changes were related to an activation of thermosensitive afferent vagal nerve fibers which were found in the esophagus, stomach and duodenum (Gupta et al., 1979; El Ouazzani and Mei, 1982; Villanova et al., 1997).

Also the MBP was slightly higher during the 30 min after cold water (cold water: +5 ± 1 mmHg; hot water: +3 ± 1 mmHg, *p* < 0.05), this apparent transient load on the heart was compensated by a decrease in HR and in the DP. The observed slightly higher rise in MBP in the first 30 min after cold water was accompanied by a greater increase in TPR and a higher decline in SkBF, which indicates peripheral skin vasoconstriction in response to cold tea ingestion. On the other hand, the increased MBP observed after hot tea was mediated through changes in CO rather than in TPR with smaller effects on SkBf, which is indicative of different autonomic mechanisms being involved. Further evidence for a different autonomic mechanism can be derived from the calculated DP, suggesting that hot tea consumption may result in a higher myocardial oxygen demand whereas DP was decreased after cold tea ingestion. Taken together, these data suggest that an unsweetened caffeinated herbal tea consumption at cold temperature (~3°C) may induce better protective effects on the cardiovascular function than at hot temperature (~55°C).

### Metabolic changes with yerba mate ingestion

Our findings revealed a rise in EE after Yerba Mate consumption, that was more pronounced with cold drink compared to hot drink (EE: +8.3% vs. +3.7% over 90 min, respectively). We observed larger increase after cold tea than previous studies where ingestion of a similar amount of cold tap water was found to increase EE by 2.9% over 90 min (Girona et al., 2014). Yerba Mate contains biological compounds including caffeine (Heck and De Mejia, 2007), a compound already described as a thermogenic agent (Chou, 1992). In the present study, the thermogenic effect of caffeinated herbal tea was more pronounced with cold drink. MacNaughton et al. (1990) also showed a greater increase in oxygen consumption associated with higher levels of catecholamines after ingestion of caffeine during exposure to cold air (5°C) compared to ambient air (28°C). An interaction between caffeine and sympathetic activity on thermogenesis (Astrup et al., 1992; Dulloo, 1993) could explain the greater EE after drinking cold tea in our study. Indeed, cold exposure increases plasma norepinephrine concentration with no effect on epinephrine whereas caffeine increases plasma epinephrine without altering norepinephrine (MacNaughton et al., 1990). Both norepinephrine (Zauner et al., 2000) and epinephrine (Matthews et al., 1990) increase EE, explaining the greater oxygen consumption after the ingestion of Yerba Mate at 3°C than at 55°C. Both a direct sympathetic stimulation (Rintamäki, 2007) and an indirect effect of circulating catecholamines (van Brummelen et al., 1986) cause vasoconstriction, can explain the greater decrease in SkBf observed after drinking cold tea in our study. Moreover, averaged over the 90 min post-ingestion, we observed a decrease in RQ after drinking cold tea but not hot tea suggesting a greater fat oxidation. Taking together, these data highlight the benefits of drinking an unsweetened caffeinated herbal tea at a cold temperature since it stimulates thermogenesis and fat oxidation and thus has the potential to influence body weight and body composition via changes in both EE and substrate utilization.

### Gender differences

Averaged over 90 min post-drink ingestion, our data highlighted only minor gender differences on the cardiovascular and metabolic response to the ingestion of a caffeinated herbal tea. Because women have in general a smaller body weight than men and because fixed amounts of caffeine and volume were given to all participants, we studied the impact of body weight on the cardiovascular and metabolic responses for both drinks. Subjects with a smaller body weight received per kg a greater amount of caffeine and volume. The lower HR observed in women does not seem to be related to the increase in MBP since we did not observe any relationship between changes in MBP and body weight, whereas a correlation was found between body weight and changes in HR. Furthermore, the increases in MBP were similar between the two drinks from 30 to 90 min, whereas there were still clear differences in HR. We speculate that the lower HR in women seems to be more related to the amount of cold fluid ingested. We are not aware of any study analyzing the gender differences on the cardiovascular and metabolic response to acute ingestion of tea. Farag et al. (2010) analyzed the cardiovascular effects of caffeine ingestion and observed no gender differences on hemodynamic responses (Rudelle et al., 2007) also observed no gender effects on the metabolic response to a beverage containing green tea catechins and caffeine.

## Conclusion

In this study, we demonstrate that ingestion of unsweetened caffeinated herbal tea at cold temperature induced a greater stimulation of thermogenesis and fat oxidation than hot tea with no increase in cardiac output work and even a decrease in myocardial oxygen demands as assessed by the DP. In this context, it is of major interest because cold caffeinated herbal tea ingestion differed from sympathomimetic drugs, whose use as antiobesity thermogenic agents is limited by their adverse cardiovascular effects and, hence, are particularly inappropriate for obese individuals with hypertension and other cardiovascular complications. Further experiments are needed to evaluate the clinical impact of unsweetened caffeinated herbal tea ingestion at a cold temperature for weight control.

## Author contributions

CM: Conceived and designed research, contributed to acquisition, analysis, and interpretation of data, drafting of the manuscript, and critical revision of the manuscript for important intellectual content; DS: Contributed to acquisition and interpretation of data and critical revision of the manuscript for important intellectual content. AD: Contributed to interpretation of data and critical revision of the manuscript for important intellectual content; J-PM: Conceived and designed research, contributed to analysis and interpretation of data, drafting of the manuscript, and critical revision of the manuscript for important intellectual content.

### Conflict of interest statement

The authors declare that the research was conducted in the absence of any commercial or financial relationships that could be construed as a potential conflict of interest.

## Abbreviations

BP: Blood pressure
BRS: baroreflex sensitivity
CW: cardiac output work
DBP: diastolic blood pressure
DP: double product
EE: energy expenditure
HF_RRI: High-frequency power components of RR intervals
HR: heart rate
LDF: laser Doppler flowmetry
MBP: mean blood pressure
RQ: respiratory quotient
SBP: systolic blood pressure
SkBf: skin blood flow
SV: stroke volume
TPR: total peripheral resistance.
